# Neurological Phenotypes of SOCS1 Haploinsufficiency: Insights from Functional and Histological Investigations

**DOI:** 10.1007/s10875-025-01958-z

**Published:** 2025-11-18

**Authors:** Serena Palmeri, Ignazia Prigione, Francesca Schena, Marie Jeanpierre, Arinna Bertoni, Federica Penco, Paola Bocca, Genny Del Zotto, Sara Massucco, Consuelo Venturi, Angelo Schenone, Gino Tripodi, Giada Recchi, Marina Lanciotti, Maurizio Miano, Caterina Matucci-Cerinic, Gianmaria Viglizzo, Riccardo Papa, Frédéric Rieux-Laucat, Roberta Caorsi, Marco Gattorno, Stefano Volpi

**Affiliations:** 1 Department of Neuroscience, Rehabilitation, Ophthalmology, Genetics, Maternal and Child Health (DINOGMI), University of Genoa, Genoa, Italy; 2 Paediatric Rheumatology and Autoinflammatory Diseases Unit, IRCCS Istituto Giannina Gaslini, Genoa, Italy; 3 Laboratory of Immunogenetics of Pediatric Autoimmune Diseases, Université Paris Cité, INSERM UMR 1163Imagine Institute, Paris, France; 4 Integrated Department of Services and Laboratories, IRCCS Istituto Giannina Gaslini, Genoa, Italy; 5 Pathology Unit, IRCCS Ospedale Policlinico San Martino, Genoa, Italy; 6 Neurology Unit, IRCCS Ospedale Policlinico San Martino, Genoa, Italy; 7 Immunohaematology and Transfusion Centre, IRCCS Istituto G. Gaslini, Genoa, Italy; 8 Hematology Unit, Department of Pediatric Hematology/Oncology, IRCCS Istituto Giannina Gaslini, Genoa, Italy; 9 Dermatology Unit, IRCCS Istituto Giannina Gaslini, Genoa, Italy

**Keywords:** SOCS1 haploinsufficiency, ALPS, Neuropathy, Multiple sclerosis, Complex regional pain syndrome

## Abstract

Suppressor of cytokine signaling 1 (SOCS1) haploinsufficiency is a recently described inborn error of immunity characterized by autoimmunity, inflammation, lymphoproliferation, and increased infection susceptibility. SOCS1, a negative regulator of cytokine signaling via the JAK/STAT pathway, explains the condition’s broad phenotypic variability. Single nucleotide polymorphisms in SOCS1 have been linked to multiple sclerosis (MS), and SOCS1 mimetics have shown efficacy in MS animal models. However, neurological involvement has not been previously reported in patients with SOCS1 insufficiency. We describe a family with a heterozygous SOCS1 variant, highlighting neurological manifestations such as MS, autoimmune encephalitis, and recurrent complex regional pain syndrome as novel features. Next-Generation Sequencing and segregation analysis were performed on PBMCs from patients and healthy donors. Functional studies included luciferase reporter assays in HeLa cells expressing the SOCS1 mutant, flow cytometry for phenotypic analysis, and gene expression profiling of the type-I interferon (IFN) signature. Intraepidermal nerve fiber density was evaluated via immunohistochemistry on skin biopsy. Genetic analysis confirmed the variant’s inheritance. Transfected cells carrying the SOCS1 variant showed increased STAT1 transcriptional activity after IFN-γ stimulation. Elevated STAT5 phosphorylation and T-cell proliferation were observed in response to IL-2. Peripheral blood revealed an elevated IFN signature during relapse. Skin biopsy showed reduced intraepidermal nerve fiber density. This report expands the clinical spectrum of SOCS1-related disorders to include neurological symptoms, emphasizing SOCS1’s critical role in regulating inflammation in the central and peripheral nervous systems.

**Supplementary Information:**

The online version contains supplementary material available at 10.1007/s10875-025-01958-z.

## Introduction

Suppressor of cytokine signalling (SOCS1) haploinsufficiency has been recently classified among monogenic autoinflammatory diseases. This genotype has been associated with a broad spectrum of symptoms, including the development of early onset multiple autoimmunity, autoinflammatory symptoms, increased susceptibility to infections, lymphoproliferation, and atopy [1–4].

The explanation for this wide range of phenotypes can be elucidated by the crucial role of SOCS1 as a negative regulator of different cytokine signaling obtained as a result upon binding with JAK1/2 and TYK2 and inhibiting their phosphorylation [5–7]. Additionally, SOCS1 acts as an intracellular adaptor for the E3 ligase complex, responsible for ubiquitinating target proteins for proteasomal degradation [8].

Consistently with the molecular mechanisms, in vitro studies have demonstrated hyperactivation of the JAK/STAT pathway in these patients, along with increased proliferation of T lymphocyte blasts after stimulation with IL-2 and IFN-γ, and a reduction in the percentage of FoxP3-positive regulatory T cells [1, 9]. Interestingly, the addition of JAK inhibitors in vitro has resulted in the normalization of these alterations [1, 3, 9]. In line with this potential pathological mechanism, a few patients have been treated with JAK inhibitors with preliminary positive results [10, 11].

In the present report we describe a family carrying a very rare heterozygous variant in the SOCS1 gene, with a history of recurrent fever, multiple autoimmune manifestations, and, as the first report in the literature, neurological involvement represented by multiple sclerosis (MS), autoimmune encephalitis and recurrent complex regional pain syndrome, with evidence of altered intraepidermal nerve fiber density on skin biopsy.

To date neurological phenotypes have never been described in individuals with SOCS1 haploinsufficiency (Table 1) [3, 12]. However, a role of SOCS1 protein in regulating central nervous system immunity and, consequently, SOCS1 alterations in the development of demyelinating pathologies have been described in MS animal models [13, 14]. Moreover, SOCS1 polymorphisms have previously been associated with increased susceptibility to multiple sclerosis development [15] and epigenetic variations of SOCS1 gene have been highlighted between MS patients and healthy individuals [16]. Furthermore, SOCS1 prevents nerve damage by regulating the inflammatory response in perineural tissues [17].

**Table 1:** Clinical features of previously reported SOCS1 haploinsufficiency patients

SOCS1 haploinsufficiency Previously reported cases	Autoimmunity	Autoinflammation	Skin disease	Infectious complications	Lymphoproliferative disease	Atopy
N of cases	19/24	13/24	14/24	10/24	7/24	11/24
% tot	79%	54%	58%	42%	29%	46%
Clinical Features	AIN (5/24) AIHA (6/24) ITP (11/24) Alopecia (1/24) AI thyroiditis (2/24) AI hepatitis (2/24) AI pancreatitis (1/24) Celiac disease (1/24) SLE (2/24) Systemic sclerosis (1/24) Raynaud’s syndrome (1/24) Myositis (1/24)	Uveitis (1/24) Fever (4/24) Arthritis (4/24) Oral ulcers (1/24) Diarrhea (1/24) MIS-C (1/24) Enthesitis (2/24) Spondylitis (1/24) Hepatopulmonary syndrome (1/24) Idiopathic pericarditis (1/24) Crohn’s disease (1/24) Chronic intestinal pseudo-occlusion (1/24)	Eczema (4/24) Psoriasis (7/24) Cutaneous lupus (2/24) Systemic sclerosis (1/24) Pyoderma gangrenosum (1/24)	Pneumonia (5/24) URI (3/24) UTI (1/24) Otitis media (1/24) HSV (1/24) Zoster (1/24) SARS-CoV2 induced inflammatory syndrome (2/24) Skin abscesses MSSA+ (1/24) Dental abscess (1/24) TBC (1/24) Aspergillus (1/24)	Splenomegaly (4/24) Hepato-splenomegaly (2/24) Lymphadenopathy (3/24) Hodgkin lymphoma (1/24)	Eczema (4/24) Allergic rhinitis (3/24) Increased IgE (2/24) Hypereosinophilia (2/24) Asthma (4/24) Dysphagia due to mild eosinophilic infiltrate (1/24) Food allergy (1/24)

*AIN *Autoimmune Neutropenia, *AIHA* Autoimmune Hemolytic Anemia, *ITP* Immune Thrombocytopenic Purpura, *AI* Autoimmune, *SLE* Systemic Lupus Erythematosus, *MIS-C* Multisystem Inflammatory Syndrome in Children, *URI* Upper Respiratory Infection, *UTI* Urinary Tract Infection, *HSV* Herpes Simplex Virus, *SARS-CoV2* Severe Acute Respiratory Syndrome Coronavirus 2, *MSSA* Methicillin-Sensitive Staphylococcus Aureus, *TBC* Tuberculosis

## Materials and Methods

### Genetic Sequencing and Segregation Analysis

Next-Generation Sequencing (NGS) panel targeting genes responsible for autoinflammatory and immuno-hematological disorders was performed on extracted DNA from Patient 1 and Patient 2’s peripheral blood mononuclear cells (PBMC) using Custom Sophia Genetics panel on MiSeq system (Illumina). Data analysis was performed using Sophia DDM software. Analysis depth at least 20x. All Pathogenic, Likely pathogenic or VUS considered of interest were confirmed by Sanger analysis, as well as for the segregation study of the variant in Patient 1 and Patient 2’s parents. Microdeletion and microduplication were identified by SNP-array.

### Peripheral Blood Samples and PBMC Isolation

Blood samples were drawn in EDTA-coated Vacutainer tubes from patient, relatives and healthy donors. Samples from healthy volunteers were collected from the Blood Bank of Istituto G. Gaslini (Genova, Italy). Peripheral blood mononuclear cells (PBMC) were isolated by Ficoll (Lympholyte-H, Cedarlane) density gradient centrifugation and stored in liquid nitrogen for further use.

### Flow Cytometry

Thawed PBMC were resuspended in RPMI 1640 with 5% FBS (Gibco) and allowed to recover for 30 min at 37° C. Cells were resuspended in staining buffer (PBS with 0,5% Bovine Serum Albumin and 2mM EDTA, Sigma-Aldrich) and stained with CD14/CD64 (Oncomark™ BD Biosciences) monoclonal antibodies (mAbs)at room temperature for 15 min. For analysis of circulating regulatory T cells (Tregs), PBMC were surface stained with Human regulatory T cell cocktail (BD Biosciences) at room temperature for 15 min. After being washed, cells were fixed and permeabilized with Foxp3 Transcription Factor Staining Buffer Set (eBiosciences) and stained with Foxp3 PE mAb or isotype matched Ig PE (eBioscience) at 4 °C for 30 min. Cell were acquired by Flow Cytometry with FACS Canto II (BD Biosciences), analysis was performed with Kaluza 2.1 (Beckman Coulter) and FACS Diva (BD Biosciences) software. To identify T helper 1(Th1) and T helper 17 (Th17) cell subsets, PBMC were stained with mAbs anti-human CD3 PerCP Cy5.5 (Sony Biotechnology), anti human CD4 FITC (BD Biosciences), anti human CD45R0 APC (eBiosciences), anti human CXCR3 PE (R&D Systems) and anti human CCR6 Pe-Cy7 (BD Biosciences). The expression of chemokine receptors was analysed gating on CD3 + CD4 + and CD45R0 + T cells.

Monocyte CD64 expression levels were calculated as the difference between CD64 geometric Mean Fluorescence Intensity (MFI) values on monocytes and geometric MFI values on lymphocytes, used as internal negative reference population.

### STAT5 Phosphorylation in Patients’ T Cells

PBMC were resuspended in serum-free medium, left untreated or treated with IL-2 (20.000 U/ml) for 15 min at 37 °C. Cells were fixed with pre-warmed Fixation Buffer (BioLegend) at 37 °C for 15 min and permeabilized with pre-chilled True-Phos™ Perm Buffer (BioLegend) at −20 °C for 1 h. Cells were then stained with CD3 PeCy7 mAb (Sony Biotechnology) and phospho-STAT5pY694 Alexa Fluor 488 mAb (BD Biosciences) for 30 min at room temperature and acquired by Flow Cytometry.

### Proliferation Assay

For T cell blasts generation PBMC were resuspended in RPMI 1640 with 10% FBS, 2mM L-glutamine, 100 U/ml penicillin and 100 ug/ml streptomycin (complete medium) (EuroClone) and seeded in CD3/CD28 mAbs coated 24 well plates for 72 h. T cell blasts were expanded in complete medium with IL-2 (100U/ml) for 10 days, then starved of IL-2 for 72 h and incubated with 5 µM carboxyfluorescein diacetate succinimidyl ester (CFSE) for 15 min at 37 °C in the dark (CellTrace™ CFSE Cell Proliferation Kit- Invitrogen). Cells were washed twice, resuspended in complete medium and seeded into 96 well plate (200.000 cells/well) in the absence or in the presence of IL-2. After 4 days culture, cells were analyzed by Flow Cytometry.

### Plasmatic Cytokine Levels

Inflammatory cytokines levels were evaluated in plasma from patients with Ella automated immunoassay platform (Bio-Techne). Simple-Plex 16 samples cartridge for the analysis of four analytes were performed in two different panel: (i) IL18-IL1RA-IL6-TNFR1(p55), (ii) CXCL10-IFNγ-IL18-CXCL9. All samples were diluted 1:2 in supplied diluent directly into the cartridge and the automated immunoassay was performed with the Simple Plex Runner Software as manufacture instruction. Normal reference ranges were established based on the analysis of plasma cytokine levels in 30 healthy donors during a phase of well-being.

### SOCS1 Expressing Plasmid and Mutagenesis

The mammalian expression vector pCMV-SOCS1-HA-tagged, used for transfection experiments, was obtained from a previous study [1]. SOCS1 mutants were generated following the protocol of the Q5 Site-Directed Mutagenesis Kit (NEB).

#### Luciferase Reporter Assays

HeLa cells were dispensed into a 96-well cell culture plate and transiently cotransfected using Lipofectamine 2000 (Life Technologies) with a luciferase reporter vector under the control of the interferon γ -activated (GAS) promoter (Promega), a Renilla control vector (Promega), and plasmids expressing either WT SOCS1, mutant SOCS1 cDNA, or a mock vector. 6 h after transfection, the cells were transferred back into medium containing 10% FBS and cultured for 24 h. Transfected cells were then stimulated (or not) with IFN-γ (103 IU/ml) for 24 h and subjected to luciferase assays with the Dual-Glo luciferase assay system (Promega). Experiments were performed in triplicate, and firefly luciferase activity was normalized against the level of Renilla luciferase activity.

#### Interferon-Signature

RNA was extracted from whole blood drawn in PAXgene tubes using PAXgene Blood RNA Kit (Qiagen, Hilden, Germany). cDNA was retrotranscribed using SuperScript^®^ VILO™ cDNA Synthesis Kit (Invitrogen, Carlsbad, California, USA). Selected IFN-stimulated gene (*IFI27*,* IFI44L*,* IFIT1*,* ISG15*,* RSAD2*,* SIGLEC1*) expression was quantified by real-time PCR using gene-specific primers and probes (Roche) with the ddCt method relatively to a healthy donor calibrator using HPRT and TBP as reference genes [18].

#### Intraepidermal Nerve Fiber Skin Biopsy Methodology

The skin samples were obtained using a 4-mm disposable punch under a sterile technique, after topical anaesthesia with lidocaine. For quantification of intraepidermal nerve fiber density (IENFD), hairy skin biopsies were obtained from the distal part of the leg (about 10 cm proximal to the external malleolus) and the lateral aspect of the thigh (about 15 cm above the patella). The specimen was fixed overnight in 2% paraformaldehyde-lysine periodate at 4 °C and then kept in a cryoprotective solution and serially cut with a freezing microtome. Free-floating 50-µm sections were obtained and incubated first with a primary antibody against the Protein Gene Product 9.5 (PGP 9.5), and then with a secondary antibody (biotinylated anti-rabbit IgG). After using the avidin-biotin ABC complex, the incubation with a substrate peroxidase kit produced a blue-gray reaction. Intraepidermal nerve fibers (IENFs) were counted at high magnification (40x) under a light microscope, and they were counted in at least three 50-µm thick sections per biopsy. Single IENFs crossing the dermal-epidermal junction were counted, while secondary branching and isolated nerve fragments within the epidermis were excluded from quantification. IENF linear density was measured per linear millimeter (IENF/mm).The fifth percentile for sex in the 20–29-year age range, which is 8.4/mm, was used as a cutoff, as no reference values for pediatric age are available [19].

#### Statistical Analysis

GraphPad Prism 8.3.0 Portable software was used for data analysis. Unpaired data from groups (HD, patients) were compared with the nonparametric Mann–Whitney U test. *P < 0.05 was considered statistically significant: **P < 0.05, **P < 0.01.

## Results

### Patients’ Presentation

Index case (P1) is a young Caucasian girl. Since the age of 3 she has experienced episodes of recurrent fever occurring twice a month, accompanied by oral aphthae, tonsillitis and lateral cervical lymphadenopathy. At the age of 9 she began to experience arthralgia and occasional mild signs of arthritis, especially during fever episodes. She developed recurrent abdominal pain, occurring both during fever and in the absence of fever, responsive to steroids, for which she underwent appendectomy at the age of 12 without symptoms resolution. Despite surgery, abdominal pain persisted, responsive only to oral steroids. Esophagogastroduodenoscopy and colonoscopy at the age of 13 revealed submucosal hemorrhages, without signs of inflammatory bowel disease. Lack of response to topical mesalazine, systemic inflammation and prompt response to steroids suggested recurrent abdominal serositis as a possible underlying condition. Of note, the patient developed multiple autoimmune conditions: celiac disease when she was 5 years old, autoimmune thyroiditis and type 1 diabetes at 8 years of age, autoimmune erosive gastritis and limbic encephalitis with positive anti-GAD antibodies in cerebrospinal fluid, presenting with epileptic seizures when she was 10 years old. She also suffered from chronic headache and experienced recurrent thrombophlebitis at the site of peripheral venous catheter, with normal coagulation parameters. The family history revealed the presence of multiple sclerosis in the father, systemic sclerosis in the paternal grandmother, and psoriasis and vitiligo in the mother.

**Fig. 1:**
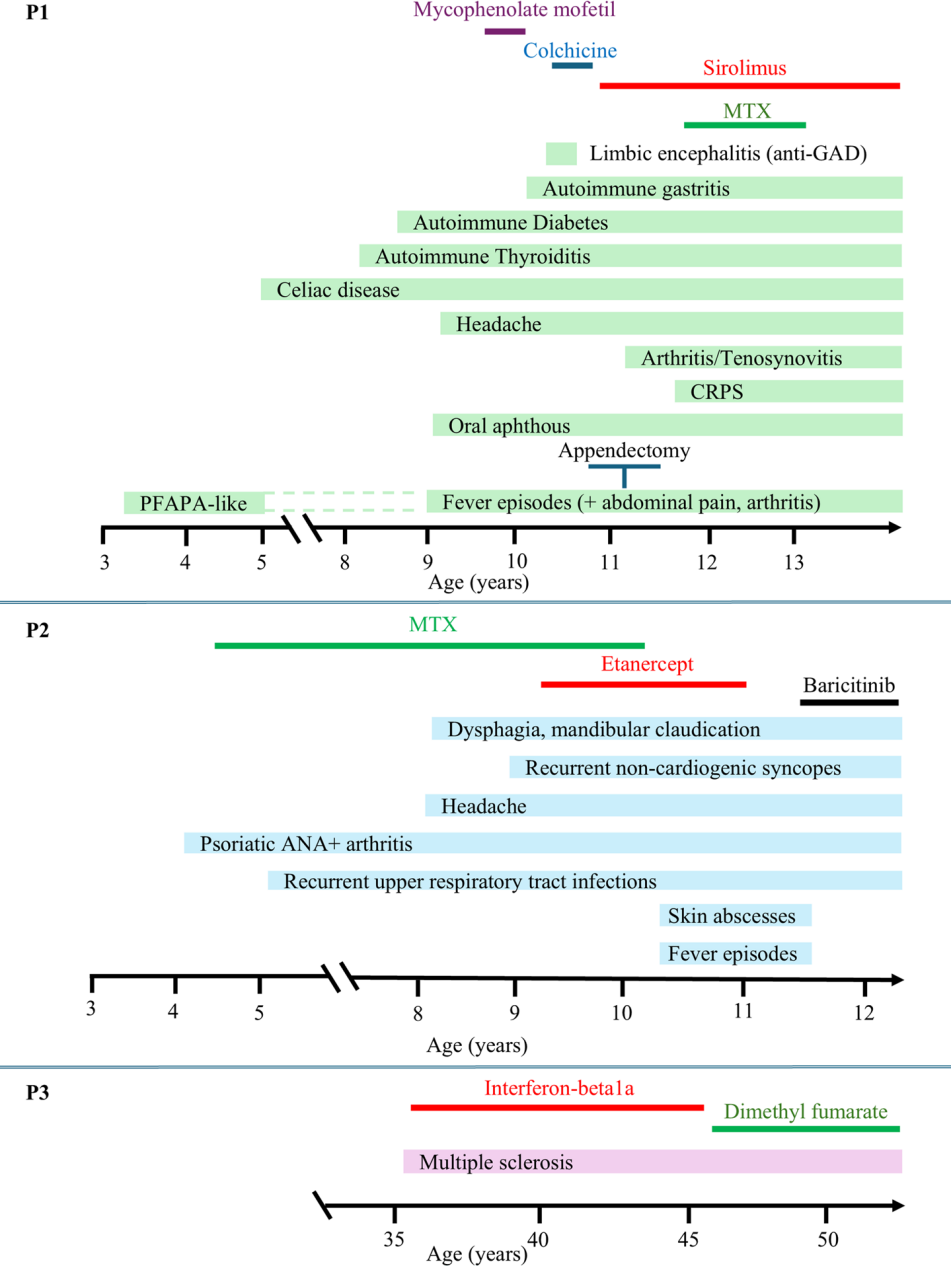
Clinical course over the years and treatments administered for Patient 1 (**P1**), Patient 2 (**P2**) and Patient 3 (**P3**) CRPS: Complex Regional Pain Syndrome; PFAPA: Periodic Fever, Aphthous Stomatitis, Pharyngitis, and Adenitis

She was firstly evaluated at our hospital at 11 years of age. Her past therapy history included colchicine (discontinued after 1 month due to gastrointestinal intolerance) and mycophenolate mofetil, which was ineffective (Fig. 1).

**Fig. 2:**
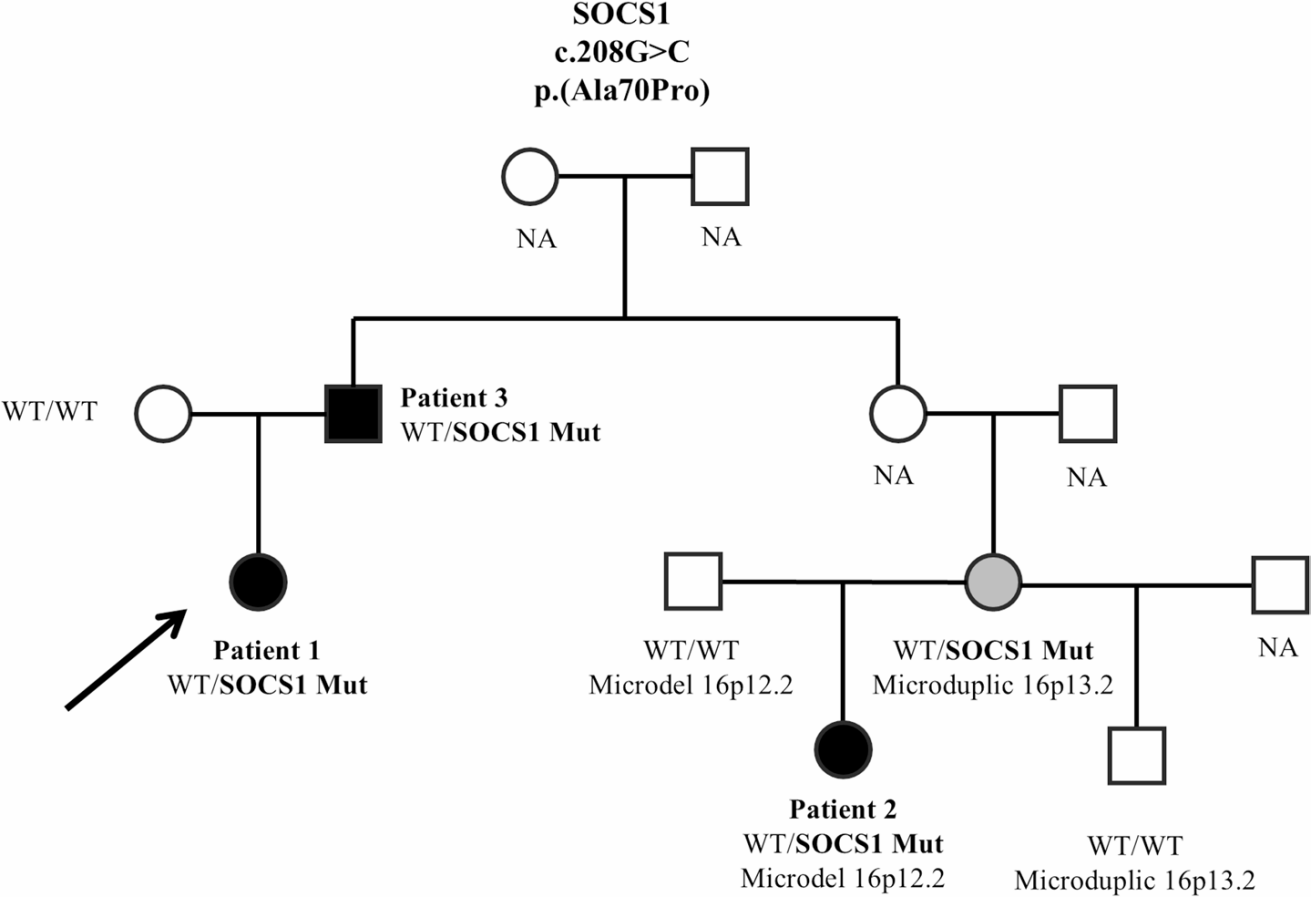
Pedigree of the Family. Affected patients with SOCS1 haploinsufficiency are represented in black, asymptomatic carrier is represented in gray. NA: not available

Blood tests showed increased inflammatory markers during flares with normalization during clinical remission, while cell blood count, C3 and C4 fractions, IgG subclasses, antibody response to vaccines, T cell proliferation in response to PHA, B cell proliferation and plasma cells differentiation, NK degranulation test and perforin expression were normal. Antinuclear antibodies were positive, while ANCA, anti-phospholipids, dsDNA, Extractable-nuclear Antigens (ENA), anti-citrullinated peptide and rheumatoid factor were negative. Lymphocyte subsets were within normal ranges except for reduction of CD19 + CD27 + cells and CD3 + CD25+/CD3 + HLA DR + ratio, mild Hyper-IgE, vitamin B12 increase (>1500 pg/mL), FAS-mediated lymphocyte apoptosis was normal (Table 2) [20]. Notably, CD3 + TCRαβ + CD4- CD8- lymphocytes resulted normal, while a slight increase was reported at 8 years of age (2.4%). Abdominal ultrasound showed multiple enlarged mesenteric lymph nodes. Considering similarities with autoimmune lymphoproliferative syndrome (ALPS) and inefficacy of MMF, rapamycin was started with a good control of systemic symptoms. A few months later, she started to display episodes of arthritis and tenosynovitis. Methotrexate (MTX) was added to rapamycin, resulting in good control of the arthritis; however, due to intolerance, it was discontinued after one year. Since the age of 12, the patient experienced eight episodes of debilitating complex regional pain syndrome (CRPS) of the upper and lower limbs, which slowly improved with rehabilitative therapy. Since an inherited immune dysregulation was suspected, a next generation sequencing panel was performed (Table S1). A very rare heterozygous *SOCS1* variant, c.208G >C, p.(Ala70Pro) was identified. The variant was inherited from her father, affected by multiple sclerosis (P3). (Fig. 2, Table S1). A maternally inherited c.1961 C >A, p.(Thr654Asn) *TNFAIP3* variant was also identified. (Table S1)

**Table 2:** Patient’s 1 (P1), patient’s 2 (P2) and patient’s 3 (P3) hematologic and Immunologic findings

	Result P1	Result P2	Result P3	Reference range
ANA	Positive, 1: 160, speckled	Positive, 1:320, homogeneous	Positive, 1:320, homogeneous	Negative
ENA	Negative	Negative	CENP-B, AMA-M2	Negative
dsDNA Ab	Negative	Negative	Negative	Negative
ANCA	Negative	NA	NA	Negative
Rheumatoid factor	Negative	Negative	NA	0–10 U/mL
CCP Ab	Negative	NA	NA	Negative
Cardiolipin Ab	Negative	NA	NA	Negative
B2 glycoprotein Ab	Negative	NA	NA	Negative
Lupus anticoagulant	Negative	NA	NA	Negative
TTG IgA	< 2 (gluten free diet)	0.9	NA	0–10 UI/mL
Endomysial	Negative (gluten free diet)	Negative	NA	Negative
TPO Ab	203	17	NA	0–115 UI/mL
TG Ab	186	15	NA	0–34 UI/mL
IgA	132	141	NA	70–400 mg/dL
IgG	969	1676	NA	700–1600 mg/dL
IgM	121	103	NA	40–230 mg/dL
IgG1	742	NA	NA	84–192 mg/dL
IgG2	159	NA	NA	10–42 mg/dL
IgG3	76.8	NA	NA	370–1150 mg/dL
IgG4	4.7	NA	NA	72–480 mg/dL
IgE	250	448	NA	0–200 KU/L
C3	171	96	NA	84–192 mg/dL
C4	34	17	NA	10–42 mg/dL
FAS-mediated lymphocyte apoptosis	Normal (61%)	Normal (56%)	NA	Negative (normal < 78%)
Lymphocytes subsets				
Total lymphocytes (absolute count)	1420	2950	900	1370–6810 cells/uL
CD3+ % (absolute count)	76.5 (1086.3)	83.9 (2475.05)	31.6 (284.40)	56–77
CD3 + CD4+ % (absolute count)	46.0 (653.2)	49.9 (1472.72)	29.5 (265.50)	31–43
CD3 + CD8+ % (absolute count)	21.2 (301.04)	27.4 (808.30)	1.9 (17.10)	20–27
CD4+/CD8+	2.1	1.8	15.1	1.4–2
CD3 + HLA DR+ % (absolute count)	4.7 (66.74)	2.4 (70.80)	NA	3.3–4.6
CD19+ % (absolute count)	9,8 (139.16)	9.4 (277.3)	39.8 (358.20)	12–16
CD16 + CD56 + CD3+ % (absolute count)	2.2 (31.24)	0.2 (5.9)	NA	2.5–3.5
CD16 + CD56 + CD3- % (absolute count)	12.3 (174.66)	5.5 (162.25)	27.5 (247.50)	12–16
CD3 + TCR gamma/delta+ % (absolute count)	7 (99.4)	6 (177.00)	NA	5–7
CD3 + TCR alfa/beta+ % (absolute count)	68.7 (975.54)	77.6 (2026.00)	NA	51–69
CD3 + TCRalfa/beta + CD4-CD8- % (DN)	1.5	0.6	NA	< 1.7
CD45R/B220+ (% on DN)	10	0	NA	NA
CD19 + CD27+ % (% on CD19 + cells)	10.3	14.8	NA	> 15
CD3 + CD25+ (% on total CD3+)	6.9	3.5	NA	NA
CD3 + CD25+/CD3 + HLA DR+	1.5	1.4	NA	1.5
CD3 + CD4 + CD25br + CD45RA-	0.8	0	NA	0.4–0.6
CD3 + CD45RA+	48.3	53.2	NA	29–39
T4 CD45RA + CD27+	14.7	56.7	NA	37–97
T4 CD45RA-CD27+	75.4	39.9	NA	0–5.8
T4 CD45RA-CD27-	3.5	3.2	NA	13–76
T4 CD45RA + CD27 + CD31+ (% on naive T cells)	14.2	45.7	NA	NA
T8 CD45RA + CD27+	61.5	74.9	NA	20–95
T8 CD45RA-CD27+	24.5	23.0	NA	9–65
T8 CD45RA-CD27-	6.6	1.5	NA	0.4–18
T8 CD45RA + CD27-	7.2	0.4	NA	4–100
Cytokines*				
IP-10/CXCL10	234	142	NA	48–514 pg/mL
IFNg	0.405	1.22	NA	0.16–5.48 pg/mL
IL-18	300	231	NA	107–372 pg/mL
MIG/CXCL9	275	1068	NA	165 − 133 pg/mL6
IL-1RA	229	327	NA	89.3-531pg/mL
IL-6	3.03	5.02	NA	0.5–7.3 pg/mL
TNFR1	1030	842	NA	576–1953 pg/mL

*Ab* antibody, *ANA* Antinucleus Ab, *ENA* Extractable Nuclear Antigens, *ds-DNA* double-stranded DNA, *ANCA* Anti-neutrophil cytoplasmatic Ab, *CCP* cyclic citrullinated peptide antibodies, TTG transglutaminase, TPO Thyroid peroxidase, *TG* thyroglobulin, *NA* not available, *DN* double negative*Cytokine levels were measured with Ellaas indicated in the Methods section; normal values are obtained from matched-age healthy donors

Three years later, we evaluated the 10-year-old second-degree female cousin of P1 (P2), for a history of ANA-positive psoriatic arthritis with onset at the age of 4. The disease required repeated intra-articular infiltrative treatment and was only partially responsive to subcutaneous MTX (suspended for intolerance) and anti-TNF treatment (etanercept). (Figures 1 and 2). Notably and similarly to P1, she presented recurrent fever episodes associated with arthralgia, occurring twice a month, together with recurrent skin abscesses from Methicillin-resistant Staphylococcus aureus (MRSA) and recurrent upper respiratory tract infections, for which she was awaiting adenoidectomy, and atopic symptoms, (atopic dermatitis in early childhood and seasonal rhinitis).

During early childhood, she exhibited delayed psychomotor development and dental anomalies. A CGH-array revealed the presence of 16p12.2 microdeletion (Table S2), present also in her asymptomatic father.

From a neurological perspective, she experienced frequent episodes of distal paresthesia and headaches, along with episodes of syncope. Extensive investigation from a cardiological standpoint resulted within normal limits.

Similarly to P1, she experienced recurrent thrombophlebitis at the site of peripheral venous catheter.

Blood tests revealed: normal blood count, slightly reduced number of CD19 + lymphocytes, increased IgG, positive ANA, mild Hyper-IgE (Table 2).

Abdominal ultrasound was normal, while cervical ultrasound demonstrated several cervical reactive lymphadenopathies.

Targeted Sanger sequencing of *SOCS1* gene revealed the same c.208G > C variant present in P1, while TNFAIP3 was WT. Considering the resistance of her arthritis to anti-TNF therapy, the potential pathogenic mechanism behind the disease and the existing clinical indication for JAK1/2 inhibitor for arthritis, the patient was switched to baricitinib, with a good clinical response.

Segregation analysis demonstrated the presence of the *SOCS1* variant in P3 (father of P1), who is affected by MS with a clinical onset at 35 years of age. He was initially treated with interferon beta-1a, then switched to oral dimethyl fumarate due to lack of efficacy (Figs. 1 and 2). Blood tests performed while on dimethyl fumarate therapy showed lymphopenia, with a reduction in T lymphocyte subsets (Table 2). Additionally, the variant was found in P2’s mother, who presents mild symptoms characterized by recurrent upper respiratory tract infections, peripheral paraesthesia, and headache. Of note, two different copy number variations were identified in this family: a 16p12.2 microdeletion found in P2 and her father, and a distinct 16p13.2 microduplication identified in P2’s mother (Fig. 2; Table S2). Neither variation encompasses the SOCS1 gene.

**Fig. 3:**
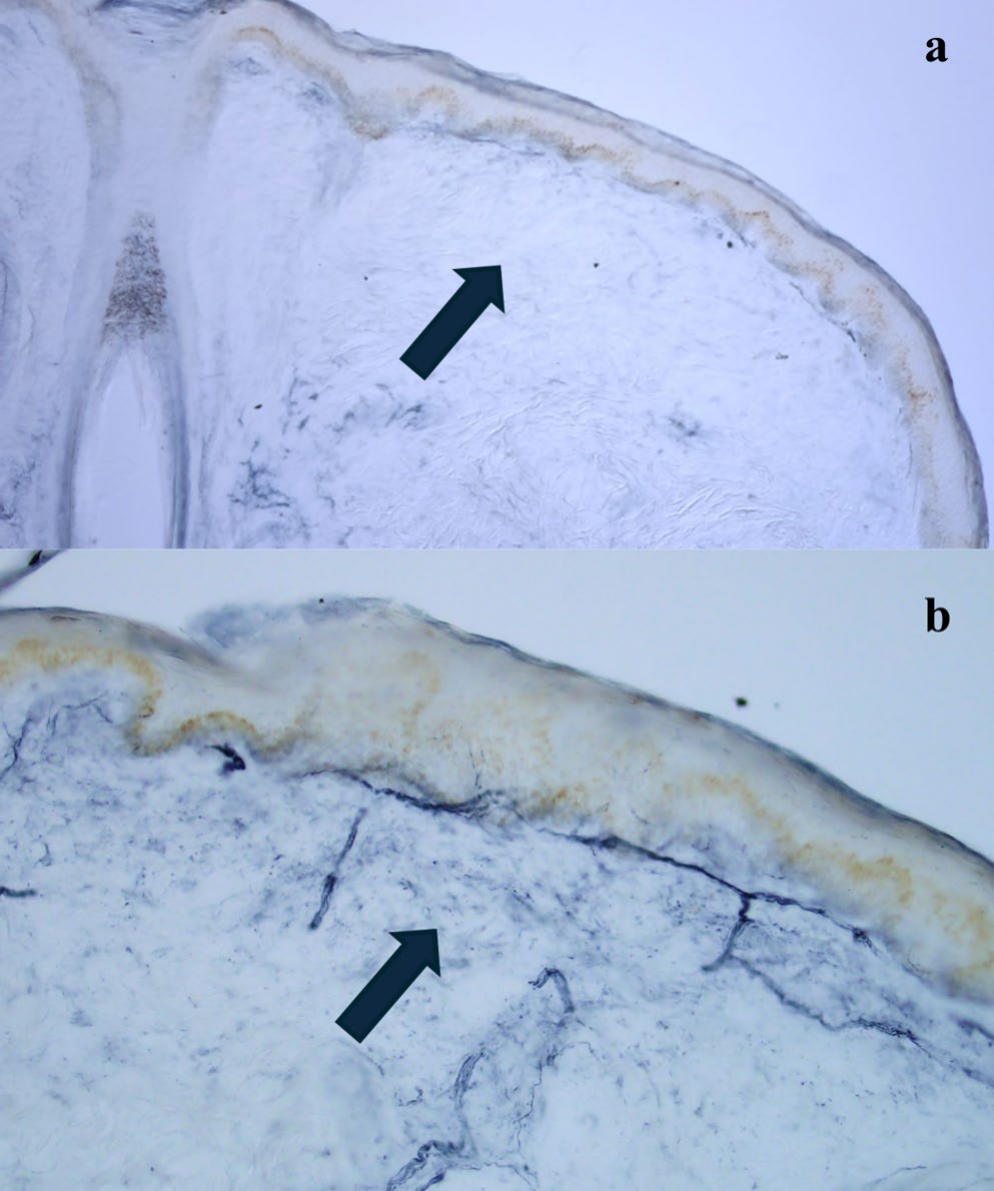
Skin biopsy, magnification 10X (**a**) and 20X (**b**). The figures show sections of the skin biopsy performed on the leg 10 cm above the external malleolus. The nerve fibers, which express the Protein Gene Product 9.5 marker, are stained blue. A normal cut-off of 8.4 mm/h was used. **a)** Patient (1) A poor representation of the subepidermal plexus and a reduction in the density of intraepidermal nerve fibers (4.02/mm) are evident. **b)** Patient (2) A better representation of the subepidermal plexus is observed, and the density of intraepidermal nerve fibers (17.02/mm) falls within the normal range considering the same cutoff

To study possible neurological alterations underlying CRPS, nerve conduction velocities were assessed and found to be normal in P1 and P2, ruling out involvement of large-caliber nerve fibers. A skin punch biopsy was performed to assess the density of small peripheral nerve fibers: a marked reduction was demonstrated in P1, who experiences recurrent episodes of CRPS and neuropathic pain with paraesthesia, while no abnormalities were observed in P2 (Fig. 3).

### Validation of ***SOCS1 ***Variant

**Fig. 4:**
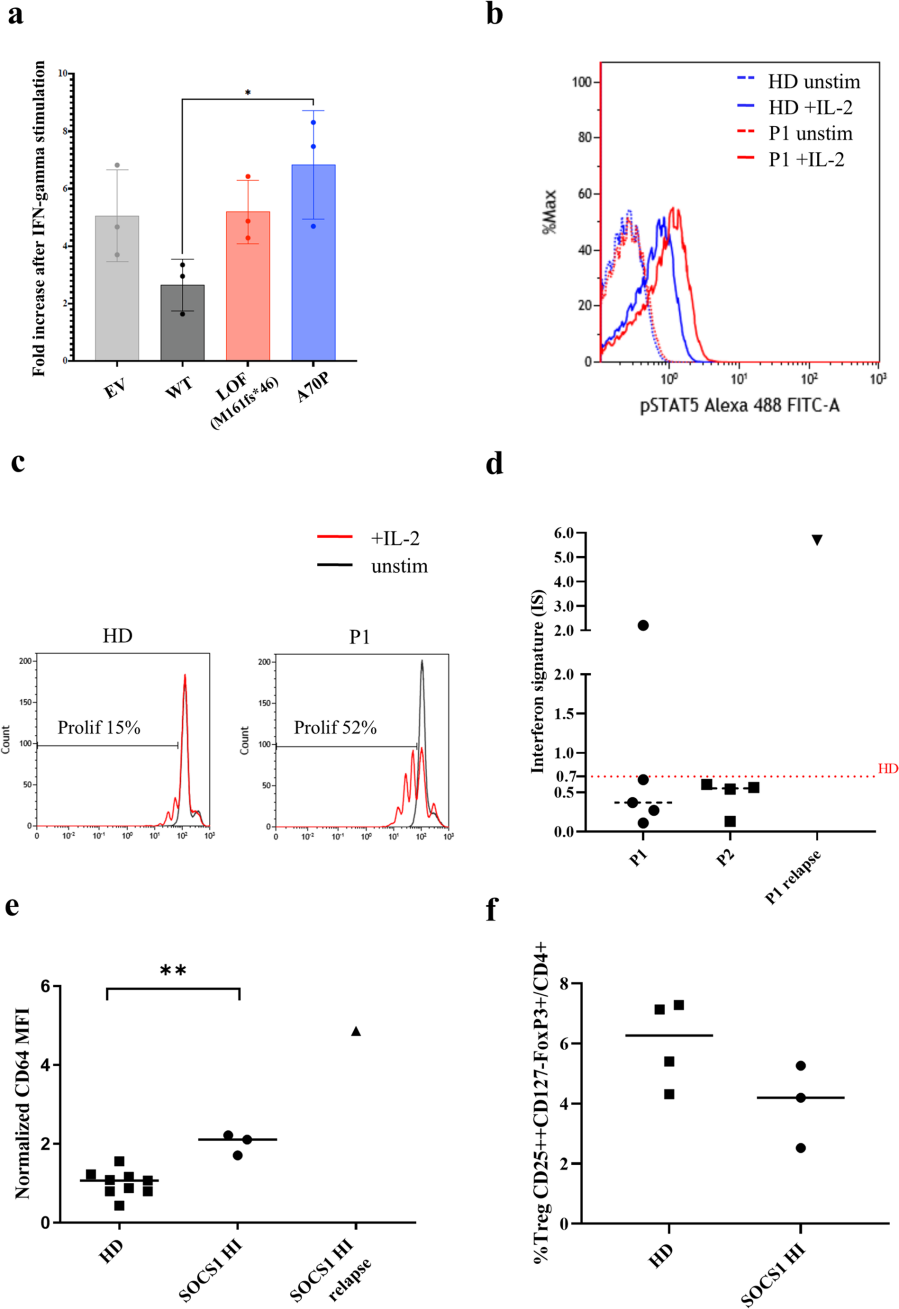
Functional validation assays(**a**) Firefly luciferase activity in HeLa cells transiently transfected with a γ -activated sequence-driven IFN-γ reporter plasmid (GAS) and expression plasmids for WT or mutant SOCS1 proteins, then stimulated with IFN-γ for 24 h. The results correspond to the fold-difference between the stimulated state and the unstimulated state. Results represent *n* = 3 independent experiments. Two-tailed p values were determined using an unpaired test. Data indicate mean with SD. *P < 0.05. (**b**) IL-2 induced STAT5 phosphorylation in T lymphocytes from patient and healthy donor. STAT5 phosphorylation levels were analyzed by flow cytometry before and after PBMC treatment with IL-2 gating on CD3 + lymphocytes. Histogram plot shows analysis for pSTAT5 in CD3 + lymphocytes from Patient1 (red) and healthy donor (HD, blue), before (dotted line) and after culture with IL-2 (20.000 U/mL) for 15 min (solid line). (**c**) Proliferation of T cell blasts from patient and HD. In vitro expanded T cell blasts, from P1 and a HD, were cultured in the presence or in the absence of IL-2 (100 U/ml) for 4 days. Proliferation was evaluated as dilution level of CFSE dye. Histogram plots show cell divisions of blasts from HD (left) and Patient 1 (right). Black line: unstimulated cells; red line: IL-2 (100 U/mL). Percentages of cells undergoing at least one division are shown. (**d**) Peripheral blood interferon signature (IS) of Patient 1 and Patient 2 in wellbeing, and of Patient 1 during relapse. The red dotted line indicates the reference range for healthy donors (HD). (**e**) Expression levels of CD64 on CD14 + monocytes from patients and HD. CD64 expression was evaluated on PBMC from 9 healthy subjects and 3 patients by flow cytometry gating on CD14 + monocytes. Data are expressed as CD64 MFI of each sample normalized to the average of CD64 MFI in healthy subjects. **P ≤ 0.01. (**f**) Frequency of circulating FOXP3 + Treg cells gated on the CD4 + T cells, analysed by flow cytometry in PBMC from healthy donors (HD) and subjects affected by SOCS1 haploinsufficiency (P1, P2, P3). MFI: Mean Fluorescence Intensity. SOCS1 HI: SOCS1 haploinsufficiency

To assess the functional effect of the mutation we analysed STAT1 transcriptional activity in HeLa cell line transfected with WT or Ala70Pro *SOCS1* plasmid. An increased STAT1 transcriptional activity was observed in cells transfected with the construct carrying the Ala70Pro *SOCS1* variant in response to IFN-γ stimulation. (Fig. 4a). As previously shown in SOCS1 haploinsufficiency, we observed increased STAT5-phosphorilation in response to in vitro IL-2 stimulation in P1 and increased proliferation of P1-derived T blasts in response to IL-2 compared to healthy donor [1] (Fig. 4b and c).

Peripheral blood type I interferon signature of P1 was sporadically positive during disease remission and it was clearly positive while in clinical relapse. (Fig. 4d) The expression of CD64, a γ interferon-stimulated gene (ISG) expressed by monocytes, was increased in CD14 + cells of patients, with a huge increase during clinical relapse in P1 [9, 11] (Fig. 4e). As previously described in both murine models [21] and patients with SOCS1 haploinsufficiency [1], we observed a trend toward a reduced proportion of CD4 + CD25 + FOXP3 + cells in the PBMCs of all three patients, although the difference was not statistically significant compared to healthy donors (HD) (Fig. 4f, Figure S1). Plasma inflammatory cytokines (IL-18, IL-1RA, IL-6, TNFRI, CXCL9, CXCL10 and IFN-γ), measured only while immunomodulatory treatment was ongoing, were within normal ranges (Table 2).

The c.1961 C > A, p.(Thr654Asn) *TNFAIP3* variant, predicted likely benign in silico (Table S1), was studied through protein expression, which was normal, and functionally by assessing NF-κB activation in patient’s primary monocytes, which also resulted normal, thus suggesting that its possible role in the disease was unlikely, also considering lack of segregation with the family phenotype (Figure S2).

To investigate a possible role of specific cell subsets in the development of neuroimmunological pathology in patient P1, we assessed the percentage of Th1 and Th17 cells in P1’s PBMCs. However, the results did not differ from those of healthy donors, with the limitation of a single measurement performed while the patient was receiving immunosuppressive treatment (data not shown).

## Discussion

**Fig. 5:**
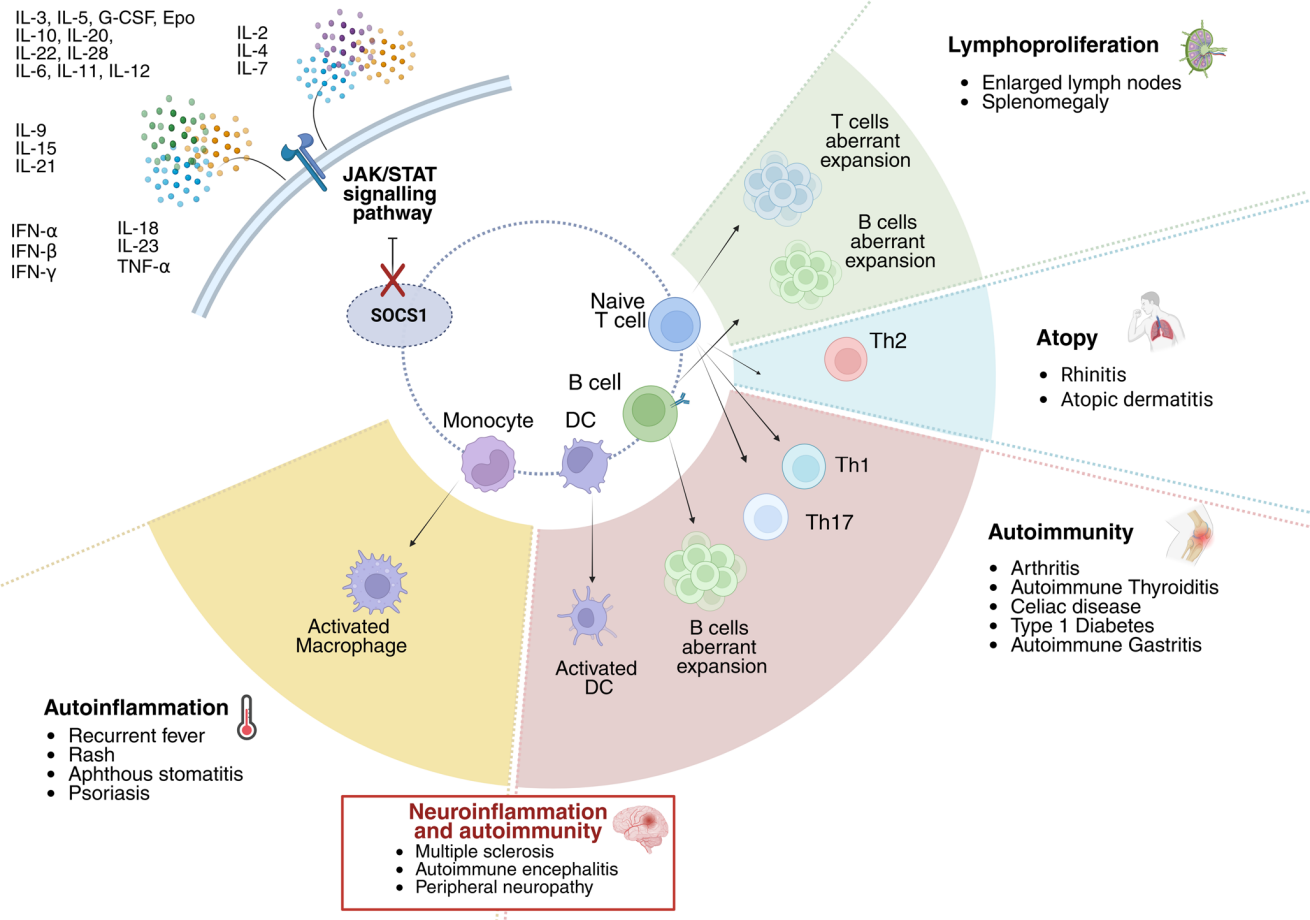
Phenotypic variability within the described family, cytokines regulated by SOCS1, and possible immunological mechanisms involved

The main novelty of our report is represented by the description of several neurological conditions associated to SOCS1 disease. Our report confirms the phenotypic variability of SOCS1 disease even in a single family carrying the same variant (Fig. 5). Two of the patients described in this study (P1, P2) were included in the multicenter analysis from the European SOCS1 registry aimed at characterizing the clinical and immunological heterogeneity of SOCS1 haploinsufficiency [4]. Interestingly, no other patient in that cohort presented neurological symptoms, therefore the present work provides the first detailed description of neurological and neuropathic manifestations in SOCS1 haploinsufficiency, including functional and histological investigations not previously reported.

The patients reported in this manuscript presented multiple sclerosis, autoimmune encephalitis, recurring episodes of complex regional pain syndrome associated with peripheral small-fiber neuropathy. Although multiple sclerosis has not yet been associated with SOCS1 haploinsufficiency, SOCS1’s role as a regulator of inflammation has been described in murine models of MS [13–16, 22, 23].

Furthermore, SOCS1 regulates the differentiation of naive T cells into T helper 1 and T helper 17 subsets, which have been identified among the primary actors initiating the inflammatory process triggering multiple sclerosis and EAE, as well as relapses [24, 25].

To date, there are no available data on the role of SOCS1 in nerve damage in humans, while data in mice demonstrate a negative correlation between SOCS1 protein levels and JAK2/STAT3 phosphorylation in injured peripheral nerves [17].

SOCS1 likely acts as a regulator of the inflammatory response in perineural tissues, thus preventing nerve damage. In addition, the expression of SOCS1 in the damaged nerve tissue has been detected in both macrophages and Schwann cells [17].

Therefore, an autoimmune cause of damage to the central or peripheral nervous system could be hypothesized, or alternatively, a direct effect of hyperinflammation due to poor regulation of the JAK-STAT pathway in tissues.

However, this interesting field is still entirely unexplored.

The second observation we can derive from the reported family regards disease variability [26].

Indeed P1, who exhibits the most severe phenotype, presented with recurrent fever, ALPS-like lymphoproliferation, and multiple autoimmune conditions since childhood, while her father (P3) is affected by adult-onset relapsing-remitting multiple sclerosis and is currently undergoing immune modulatory treatment. P2, a second-degree cousin of P1, presented with recurrent fevers and ANA-positive psoriatic arthritis, unresponsive to treatment with methotrexate and etanercept, but also concurrently exhibits chronic rhinitis and recurrent upper respiratory tract infections, while her mother, who carries the variant, shows very mild symptoms characterized by chronic rhinitis, recurrent upper respiratory tract infections, transient paraesthesia in the lower limbs.

Twenty-four patients with SOCS1 haploinsufficiency have been extensively reported in the literature so far, as a case-by-case description. The most common phenotype includes autoimmunity, often multiple and with an early onset ranging from hematological conditions to gastrointestinal disorders-such as celiac disease, arthritis and multi-organ syndromes like systemic lupus erythematosus. Interestingly, we describe for the first time the presence of autoimmune gastritis in SOCS1 haploinsufficiency [27].

The second most common group of symptoms is represented by cutaneous involvement of which psoriasis, also present in P2, is the most frequent.

The third group is represented by autoinflammation, with fever being the most prevalent [1, 8, 11, 12].

For this reason, it is important to raise awareness among clinicians to consider SOCS1 haploinsufficiency in patients with recurrent fevers who develop other complications over time, especially multiple autoimmunity.

The case of P1 is particularly illustrative of how a pattern of recurrent fever resembling Periodic Fever, Aphthous stomatitis, Pharyngitis, and Adenitis (PFAPA) can evolve into such a complex scenario over the span of approximately 10 years.

Lymphoproliferation has also been described as a manifestation of SOCS1 haploinsufficiency, in one case evolving in Hodgkin’s lymphoma. At present, the overall risk of malignancy in this condition is not known [1, 8, 27].

This significant phenotypic variability can be explained by the versatility of SOCS1 role as a regulator of the JAK/STAT pathway downstream of several cytokines including type I and type II interferons, as well as IL-2, IL-4, IL-6, IL-12, IL-15, IL-18, IL-23 and TNF-alpha. Furthermore, SOCS1 serves as a critical regulator in the response of Toll-like receptors, a class of cellular receptors involved in pathogen detection and activation of the innate immune response. It is therefore important to highlight how the coexistence of autoimmunity, autoinflammation, and atopy can be explained by the versatility of SOCS1 in regulating the inflammatory response [8, 27].

However, the family we report, suggests the existence of disease modifiers that might be genetic or environmental, able to determine a different clinical presentation despite the same SOCS1 variant.

Although data are still insufficient to assess the efficacy of JAK inhibitors in this emerging inborn error of immunity, the heterogeneous clinical presentation and the wide range of molecular mechanisms involved may suggest that a combined therapeutic strategy guided by the main symptom presented by the patient, maybe more indicated rather than a one-fits-all approach. For example, in cases with predominant lymphoproliferative symptoms, it would be useful to consider medications such as mycophenolate mofetil or rapamycin, possibly combined with an anti-cytokine biologic therapy to control the inflammatory component. To better manage the autoimmune component, it might be helpful to attempt infusions of immunoglobulins at an anti-inflammatory dose or, in more extreme cases with a lupus-like component, anti-CD20 medications have been reported [11]. However, therapy response and side effects of these approaches are currently unknown and will deserve dedicated studies. Finally, in recent years, SOCS1-mimetic peptides, such as SOCS1-KIR, have been developed. These peptides mimic the structure of SOCS1 and act as pseudosubstrates for JAK1, JAK2, and TYK2, but not JAK3. By doing so, they replicate SOCS1’s inhibitory function on these kinases and could serve as potential therapeutic agents in cases where SOCS1 protein is dysfunctional. This approach might represent a possible targeted therapy in the future, able to rescue the functional defect observed in this disorder [28, 29].

## Supplementary Information

Below is the link to the electronic supplementary material.

Supplementary Material 1 (DOCX 713 KB)

## Data Availability

The data are available from the corresponding author upon reasonable request.

## Abbreviations

ALPS: Autoimmune Lymphoproliferative Syndrome
ANA: Anti-Nuclear Antibodies
ANCA: Anti-Neutrophil Cytoplasmic Antibodies
APC: Allophycocyanin
CFSE: Carboxyfluorescein Succinimidyl Ester
CNS: Central Nervous System
CRPS: Complex Regional Pain Syndrome
DNA: Deoxyribonucleic Acid
EDTA: Ethylenediaminetetraacetic Acid
ELISA: Enzyme-Linked Immunosorbent Assay
FACS: Fluorescence-Activated Cell Sorting
FBS: Fetal Bovine Serum
FoxP3: Forkhead Box P3
IFN-γ: Interferon-gamma
IL: Interleukin
IENFD: Intraepidermal Nerve Fiber Density
JAK: Janus Kinase
JIA: Juvenile Idiopathic Arthritis
MS: Multiple Sclerosis
MFI: Mean Fluorescence Intensity
NK: Natural Killer
NGS: Next-Generation Sequencing
PBMC: Peripheral Blood Mononuclear Cells
PBS: Phosphate-Buffered Saline
PCR: Polymerase Chain Reaction
PHA: Phytohemagglutinin
PMF: Polymyalgia Rheumatica
SOCS1: Suppressor of Cytokine Signaling 1
STAT: Signal Transducer and Activator of Transcription
TCR: T-Cell Receptor
Tregs: Regulatory T Cells
TNF: Tumor Necrosis Factor
TYK: Tyrosine Kinase
RPMI: Roswell Park Memorial Institute Medium
SNP: Single Nucleotide Polymorphism
