# Supplementary material for: Neurological Phenotypes of SOCS1 Haploinsufficiency: Insights from Functional and Histological Investigations
Source: J Clin Immunol. 2025 Nov 18;45(1):165. doi: 10.1007/s10875-025-01958-z (PMC12628480; doi:10.1007/s10875-025-01958-z)
Supplement: Supplementary file 1 — Supplementary Material 1 (DOCX 713 KB) [file 10875_2025_1958_MOESM1_ESM.docx]

**Supplementary materials**

**Neurological phenotypes of SOCS1 Haploinsufficiency: insights from functional and histological investigations**

Serena Palmeri^1,2^, Ignazia Prigione^2^, Francesca Schena^2^, Marie Jeanpierre^3^. Arinna Bertoni^2^, Federica Penco^2^, Paola Bocca^2^, Genny Del Zotto^4^, Sara Massucco^1^, Consuelo Venturi^5^, Angelo Schenone^1,6^, Gino Tripodi^7^, Giada Recchi^2^, Marina Lanciotti^8^, Maurizio Miano^8^, Caterina Matucci-Cerinic^1,2^, Gianmaria Viglizzo^9^, Riccardo Papa^2^, Frédéric Rieux-Laucat^3^, Roberta Caorsi^1,2^, Marco Gattorno^2^, Stefano Volpi^1,2^

**Supplementary table 1 (Table S1). Gene variants found in P1.**

| **Gene** | **Variant** | **Zygosity** | **ID SNP** | **Frequency  (GnomAD 2.0.1)** | **Frequency  (GnomAD v4.1.0)** | **CADD (GRCh38-v1.7)** | **Varsome v12.6.1** | **ClinVar** |
| --- | --- | --- | --- | --- | --- | --- | --- | --- |
| SOCS1 | c.208G>C, p.(Ala70Pro) | Het. | - | - | 0.000072% | 19.91 | VUS | - |
| CASP10 | c.1502C>T, p.(Pro501Leu) | Het. | rs148939095 | 0.12% | 0.05762% | 24.70 | Benign | Conflicting (VUS:1 LB:1 B:2) |
| DDX41 | c.451T>C, p.(Tyr151His) | Het. | rs747442017 | 0.0016% | 0.001115% | 21.90 | Likely Benign | VUS |
| TNFAIP3 | c.1961C>A, p.(Thr654Asn) | Het. | rs780049410 | 0.00080% | 0.0001239% | 13.45 | Likely Benign | - |
| ARPC1B | c.107A>T, p.(Glu36Val) | Het. | rs767687212 | 0.00040% | 0.0001859% | 22.60 | Likely Benign | - |

Abbreviations. CADD: Combined Annotation Dependent Depletion score; Het.: Heterozygous; ID: Identifier Single Nucleotide Polymorphism; LB: Likely Benign; VUS: variant uncertain significant.

The table lists all the variants of interest identified in the proband (P1). Evaluation of population frequency and pathogenicity prediction scores was used to exclude the CASP10 variant from our diagnostic hypotheses. Although predicted as benign, the identified rare variants were subjected to further diagnostic investigation. TNFAIP3 gene was analysed through family segregation studies, which revealed maternal inheritance, TNFAIP3 protein expression on PBMCs from both the proband and the mother, along with an NF-kB functional assay in both individuals, which yielded normal results (Supplementary Figure 1). The rare variant in ARPC1B was assessed through a functional test, which also yielded normal results (data not shown).

**Supplementary Table 2 (Table S2). Copy number variations identified in the family.**

| Microdeletion 16p12.2 | 16-10071604-10525165 |
| --- | --- |
| Microduplication 16p13.2 | 16-21771016-22435811 |

The microdeletion and microduplication present in the family do not affect *SOCS1* gene.

**Supplementary Figure 1 (Figure S1). Gating strategy for Treg. cells identification by flow citometry**

**
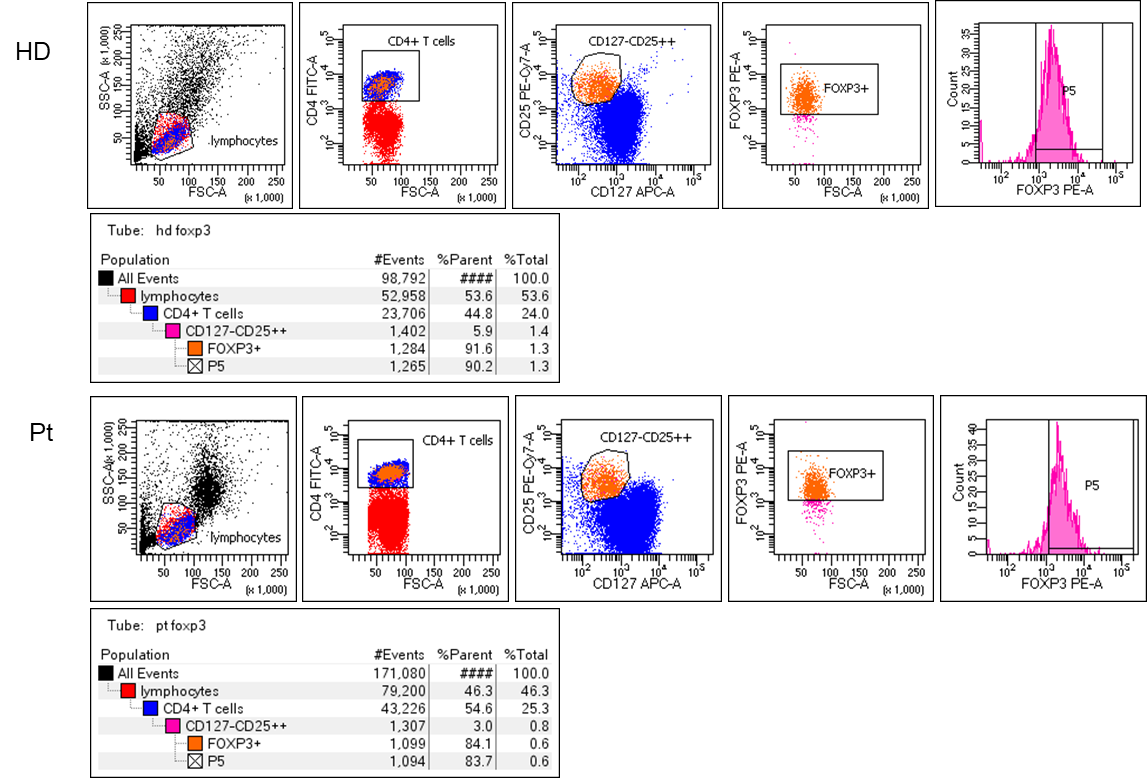
**

**Supplementary Figure 2 (Figure S2). A20 expression and pNF-κB nuclear translocation in P1 and P1’s mother.**


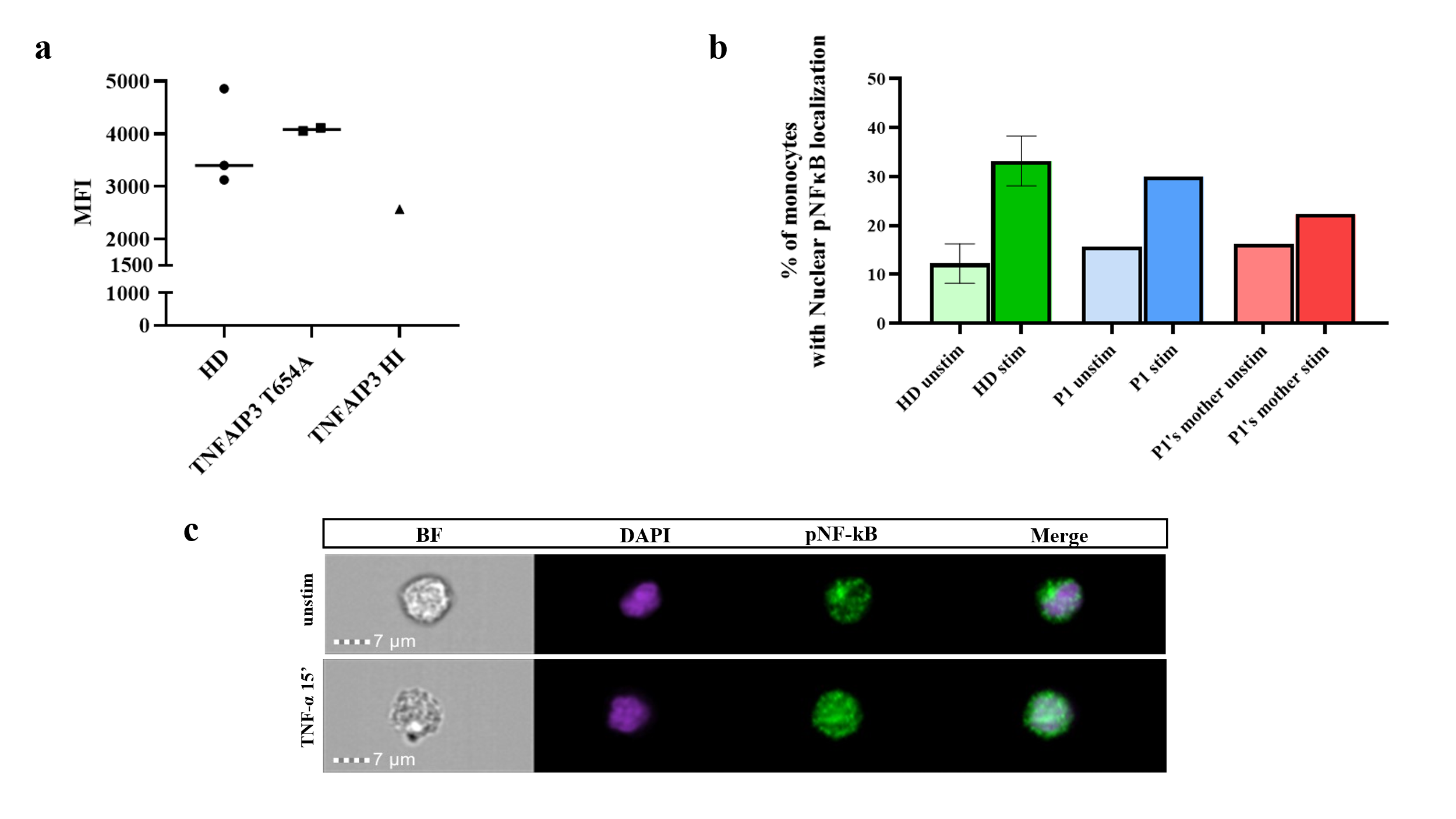


**a.** A20 expression was evaluated on PBMC from 3 healthy donors (HD), one patient affected by TNFAIP3 haploinsufficiency (TNFAIP3 HI; c.1068del; p.(Trp356Cysfs*30)), P1 and her mother (TNFAIP3 T654A) by flow cytometry. Data are expressed as difference between the geometric MFI values of A20 and the geometric MFI values of Isotype control.

**b.** p NF-κB nuclear translocation was evaluated on monocytes from 3 healthy donors (HD), P1 and P1’s mother by Imaging Flow Cytometry. Analysis was performed before (unstim) and after PBMC stimulation with TNF-α. (stim). Monocytes were selected on the basis of their Area vs SSC (side scatter, or darkfield, derived from the 786 nm laser) characteristics. Nuclear localization of p NF-κB was assessed using the “Similarity Score” feature by IDEAS® software. The percentage of monocytes exhibiting nuclear expression of pNF-κB is shown.
**c.** Analysis of pNF-kB expression and nuclear translocation. ImageStream X Mark II imaging flow cytometer was used to analyze pNf-kB nuclear relocalization after PBMC treatment with TNF-α . Monocytes were selected based on their Area vs SSC (side scatter, or darkfield, derived from the 786 nm laser) characteristics. Nuclear localization of pNF-κB was assessed using the “Similarity Score” feature in IDEAS® software. A representative image of pNF-kB subcellular localization is shown. BF: brightfield image; DAPI: nuclear image, purple; pNF-kB: green; Merge: merged images for the nucleus with pNF-kB staining.

**Supplementary Methods**

**Flow cytometry**

For A20 expression analysis, PBMC were fixed and permeabilized using BD Cytofix/Cytoperm™ (BD Biosciences) according to manufacturer’s instruction. Cells were left in permeabilization buffer, stained with anti- A20 mAb (#59A426 Invitrogen) at room temperature for 30’and then with Alexa-488 Goat anti-Mouse IgG (Invitrogen) at room temperature for 30’. Cell were acquired by Flow Cytometry. A20 expression level was evaluated as the difference between the geometric MFI values of A20 and the geometric MFI values of Isotype control.

**pNF-kB nuclear localization by Imaging Flow Cytometry**

**Staining.** PBMC were resuspended in serum-free medium, left untreated or treated with TNF-α (100 ng/ml) for 15 minutes at 37°C. Cells were fixed with pre-warmed Fixation Buffer (BioLegend) at 37°C for 15 min and permeabilized with pre-chilled True-Phos™ Perm Buffer (BioLegend) at -20°C for 1hr. Cells were stained with Phospho NF-kB p65 (Ser536) mAb (#93H1- Cell Signaling Technology) at RT for 30’ and then with Alexa-488 AffiniPure F(ab’)_2_ Goat anti-Rabbit IgG (Jackson ImmunoResearch) at RT for 30’. Before cell acquisition, DAPI (Invitrogen) was added to each sample.

**Acquisition.** Imaging data were acquired using an ImageStream Mark X II imaging flow cytometer equipped with 4 lasers (405 nm, 488 nm, 642 nm, and 786 nm) and a 20x, 40x, 60x magnification system. Cells were acquired at 60x magnification based on focus (Gradient RMS of brightfield) and physical characteristics (Area vs Aspect Ratio of brightfield). Acquired events were analyzed by IDEAS® software (v. 6.2.183, Amnis/Cytek).

**Compensation.** Single-color controls were acquired for each fluorochrome (500 events per control) and used to generate a compensation matrix in in IDEAS® software.

**Analysis.** Acquired events were analyzed by IDEAS® software (v. 6.2.183). Monocytes were selected on the basis of their Area vs SSC (side scatter, or darkfield, derived from the 786 nm laser) characteristics. Nuclear localization of pNF-κB was assessed using the “Similarity Score” feature by IDEAS® software. Similarity Score, a log-transformed Pearson's correlation coefficient, quantifies the pixel intensity correlation between pNf-κB and DAPI within the nuclear mask images (which are based on DAPI staining). Lower SS values indicate predominantly cytoplasmic pNF-κB, while higher values signify nuclear localization.
